# Dimensions of wellbeing and recognitional justice of migrant workers during the COVID-19 lockdown in Kerala, India

**DOI:** 10.1057/s41599-023-01687-x

**Published:** 2023-05-05

**Authors:** Mishal Alice Mathews, Geert De Neve, Sonja Ayeb-Karlsson

**Affiliations:** 1 United Nations University- Institute for Environment and Human Security (UNU-EHS), Bonn, Germany; 2 School of Global Studies, University of Sussex, Brighton, UK; 3 Institute for Risk and Disaster Reduction (IRDR), University College London (UCL), London, UK

**Keywords:** Development studies, Anthropology

## Abstract

The lockdown of March 2020 in India witnessed one of the largest movements of migrants in the country. The state of Kerala was quick and efficient in responding to the challenges posed by the lockdown on its migrant population and in supporting its ‘guest workers’. While many studies have researched the material resources of migrants during the pandemic, such as income and food, few have investigated the subjective measures and emphasised the lived experiences of migrant workers. Drawing on the Wellbeing in Developing Countries (WeD) approach which examines three dimensions of wellbeing, namely, (a) material, (b) relational and (c) subjective wellbeing, this article focuses on the mental health and wellbeing experiences of migrant workers during the first lockdown in Kerala. By deploying these wellbeing dimensions, the study looks at how migrant workers perceived and experienced the various interventions put in place by state and local governments, as well as voluntary initiatives aimed at supporting them. The study elaborates around migrants’ relations of love, care, and trust, and their reasons to remain in Kerala or return home during the lockdown. The study found that a paradigm shift, where ‘migrant workers’ are becoming ‘guest workers’, was at the forefront of the captured narratives. The key findings in this way contribute to the understanding of migrants’ lived experiences, wellbeing, and perceptions of the different lockdown interventions. We argue that an increased attention to subjective factors helps us understand migrant needs at times of crisis through their lived experiences and thereby enhances policy planning for disaster preparedness.

## Introduction

Originally, India’s first national lockdown in March 2020, as a response to the spread of COVID-19, had a more devastating impact than the virus itself. The lockdown paralysed rural life and the existing socio-economic fabric of society shaped the diversity of outcomes. For example, access to food was highly unequal as members of higher castes managed to store food in bulk just before the lockdown (Carswell et al., 2020). Owing to the largely informal nature of India’s economy, the lockdown amounted to a significant shutdown of economic activities (Raju et al., 2021, p. 1).

According to the World Bank (2020, p. 27), 40 million internal migrant workers in India were affected by the COVID-19 pandemic and the subsequent lockdowns. This included losing their jobs and being unable to collect their wages or make arrangements to go back to their villages (Jan Sahas, 2020, p. 6). The closing down of industries and markets led to the loss of their only source of income for millions of people (Raju et al., 2021, p. 3) and highlighted the fragility of the livelihoods and wellbeing of migrant workers (University of East Anglia, 2020). Many ended up trapped or stranded in their destination city (Raju et al., 2021, p. 3; Ayeb-Karlsson et al., 2018, p. 557), and were unable to pay rent and maintain physical distancing in small and crowded accommodations (Jan Sahas, 2020, p. 6; Rajan and Bhagat, 2021, p. 232; Aajeevika Bureau, 2020, p. 9).

The country witnessed troubling sights of migrant workers walking thousands of kilometres home (The Hindu, 2020; Pandey, 2020). The primary concern for many migrant workers with the implementation of lockdown was a safe return to their families (Jesline et al., 2021, p. 7). The Central Government informed the Supreme Court of India in April 2020 that almost 600,000 migrant workers had walked back to their villages (Business Standard, 2020) and many of them faced shortages of food and money, often resulting in deaths due to hunger, dehydration and exhaustion (Jesline et al., 2021, p. 7; Mishra and Sayeed, 2020, p. 46). The loss of jobs and income, social isolation and other insecurities deteriorated the mental health of migrant populations and was expected to worsen in post-lockdown periods (Mishra and Sayeed, 2020, p. 48). Concerns about employment, hunger and poverty, money, and children’s education were psychological stressors that the migrant workers experienced daily and that impacted them deeply (Duggal et al., 2022 p. 8220; Kumar et al., 2020, p. 3).

Even though states across India had only had a few hours to prepare for the lockdown, Kerala was relatively quick and efficient in responding to the situation and addressing the needs of its ‘guest workers’ (Peter et al., 2020, p.1065). There is a large influx of migrant workers to Kerala owing to high wage levels, a lack of semi-skilled and unskilled local labour following high levels of out-migration to Gulf countries, and the unwillingness of the youth in the state to perform unskilled work (Mohan, 2017, p. 15; Martin, 2021; The Hindu, 2020(b)). The Census of India in 2011 revealed that Kerala is one of the main destination states for internal migrants in India (Rajan and Bhagat, 2021, p. 234). According to Saika (2015, p. 29), this migration does not constitute a shift in the nature of employment for migrant workers, but a shift towards higher income levels. The way that the Kerala government constituted the Working Group on Labour Migration in its 13th Five-Year Plans (2017–2022) reflected the concern of the government for the welfare of its inter-state migrant workers (Peter et al., 2020, p. 1069). During the first lockdown, The Week (2020) reported that Kerala was setting an example for other states with its exceptional treatment of 'guest workers'. Peter et al. (2020, p. 1065) opined that the welfare measures and government interventions in Kerala for inter-state migrant workers were exemplary and promising. Rajan and Bhagat (2021, p. 236) noted that Kerala provided a template for addressing the issues of migrants as well as other vulnerable groups as it became the only state to announce a comprehensive package of Rs. 200 billion to cater to the needs of migrant workers even before the announcement of the national lockdown and the central government’s assistance scheme. The World Health Organisation identified early preparation, systematic investment in strengthening health infrastructure, testing and containment strategy, isolation of high-risk contacts, risk communication and community engagement, and providing psycho-socio support as the reasons for Kerala’s effective response to the situation and as lessons to be learned from the state (WHO, 2020).

On 25 March 2020, the Kerala government announced the opening of ‘Community Kitchens’ to supply meals free of cost or at a very subsidised rate during the lockdown (Kudumbashree, 2020; The News Minute, 2020; The Business Line, 2020; Shibu, 2020). 69 per cent of India’s government-run relief camps for migrant workers were in Kerala (Bloomberg Quint, 2020). Meanwhile, the District Intervention System for Health Awareness (DISHA), a telephone helpline, assisted migrant workers in getting checked by a doctor (Sasidevan et al., 2021, p. 57; GoK, 2020; Athira, 2020; Martin, 2021; Varma, 2021). The success of Kerala in this regard has been accredited to numerous factors. Choolayil and Putran (2021, p. 17), for example, argued that a combination of social and medical interventions took place alongside a participatory approach. Chathukulam and Tharamangalam (2020) gave credit to the participatory mode of governance in Kerala and the state’s excellent health infrastructure. Rao et al. (2020, p. 1658) explained that the social policy framework of destination states was quintessential in shaping the experiences of migrant workers during the lockdown, and they identified Kerala’s better social infrastructure and its legal and policy framework, which identified migrant workers as ’guest worker’s, as contributing to the state’s effective responses. The role of local self-government institutions, mainly panchayats, in responding to the needs of migrant workers and the challenges posed by the pandemic has been emphasised too. According to Dutta and Fischer (2020, p. 8–10), panchayats were able to identify those requiring social protection and to implement social support measures.

There are various studies on the success of Kerala in addressing the needs of migrant workers and on the various contributing factors or government interventions. Our literature search, however, identified a lack of empirical accounts examining the lived experiences of migrant workers during the lockdown. It is important to understand how migrant workers perceived their wellbeing (Ayeb-Karlsson, 2020, p. 103) and changes to their life during the lockdown as well as the various government schemes, interventions and relief measures aimed at them. Socio-economic inequality, poverty, and forms of mistreatment that impacted mental and physical health have a bearing on people’s vulnerability and ability to respond to stress (Raju et al., 2021, p. 2; Ayeb-Karlsson, 2020; Ayeb-Karlsson et al., 2020). In their study, Kumar and Pramod (2016, p. 128–130) note that migrant workers in Kerala experienced considerable levels of alienation, deprivation, stress, depression and anxiety. Following this, our article will study the mental health and wellbeing experiences of migrant workers during the lockdown in Kerala. The analysis contributes to the understanding of lived experiences and wellbeing perceptions of migrant workers and of various interventions aimed at them during the lockdown. Our study shows why subjective measures should be central to an understanding of migrant experiences during the pandemic and how to enhance policy planning. Thus, the article contributes to knowledge of migrant vulnerability and disaster preparedness, through a wellbeing approach based on the Wellbeing in Developing Countries (WeD)^1^ framework.

In the following sections, we discuss the theoretical framework deployed in this article. Firstly, we draw on the WeD framework (White, 2010) to highlight the importance of considering subjective measures in addition to objective ones while planning development policy. We analyse various dimensions of migrant workers’ wellbeing followed by a discussion of the importance of wellbeing and its relevance to policy making. Secondly, we build on the concept of recognitional justice to help us draw attention to the diversities of experiences, identities, and values of those affected and to consider how a sensitivity to difference can be integrated into policy discourses and design.

## Theoretical framework

Drawing on the work of the Wellbeing in Developing Countries Research Group (WeD), White (2010, p. 157) put forward a framework for analysing wellbeing in development practice that views wellbeing as a social process with material, relational and subjective dimensions. The three dimensions are inter-related and co-constitutive. This study draws on this framework as it contains a more holistic perspective of wellbeing and, most importantly, is centred around the person, their perceptions and priorities. This allows for attention to be given to not just objective, quantifiable measures, such as income and nutrition, but also the subjective aspects of how people perceive and experience their health, income and wellbeing.

The three key wellbeing dimensions are the material, relational and subjective (see Table 1). The material dimension consists of assets, welfare and standards of living. The relational dimension is made up of two spheres—the social and the human. While the social sphere includes social relations and access to public goods, the human sphere includes capabilities, attitudes to life and personal relationships. The subjective dimension also contains two aspects—one, people’s perceptions of their material, social and human positions, and second, cultural values, ideologies, and beliefs (White, 2010, p. 161).

**Table 1:** Summarisation of the three dimensions of wellbeing.

Dimension	Concerns	Includes
Material	(i) Practical welfare (ii) Standards of living	• Income, wealth, assets • Employment and livelihoods • Levels of consumption
Relational	(i) Social relations and access to public goods (ii) Human-capabilities, attitudes to life and personal relationships	• Relations of love and care • Networks of support and obligations • Relations with the state: welfare, politics and law • Cultural, social, political and identities • Violence, conflict and (in)security • Access to services and amenities • Environmental resources
Subjective	(i) People’s perception of their material, social and human positions (ii) Cultural values, ideologies and beliefs	• Hopes, fears and aspirations • Self-concept and personality • Trust and confidence • Levels of (dis)satisfaction with health, information, skills, services • Religious faith

The material dimension is the most familiar to development approaches (White, 2010, p. 163). However, a subjective understanding of wellbeing calls for a focus on subjective aspects or people’s own perceptions of the material, social and human dimensions of their wellbeing.

This article also deploys the concept of recognitional justice to analyse migrant wellbeing during the lockdown. Recognitional justice enables us to acknowledge social and cultural groups within the prism of social justice (Guo, 2010, p. 164). It extends the potential of social justice beyond the narrow focus of material and economic goods to include social goods such as opportunities, positions and institutional inequities. Recognition also calls for an understanding of the processes that determine distributive injustices (Young, 1990) and is based on the principles of love, equality and social esteem (Honneth, 2004, p. 355, 358). Recognition implies equal treatment of all social identities, acknowledgement of diverse participants and sensitivity to differential experiences among affected people and their participation in policy creation (Chu and Michael, 2019, p. 142). A lack of this can result in the cultural domination of some, the being rendered invisible of others, and the reproduction of routine stereotyping (Fraser, 1997). Recognition is socially constructed, contentious and context dependent, and enables critical evaluation of how differences in sociocultural identities, values, and behaviours are recognised and integrated into policy discourses (Chu and Michael, 2019, p. 141).

The concept of recognitional justice, with its focus on social goods, institutional inequities, and differential experiences of those affected complements the WeD framework of wellbeing. It can inform theorists and policy makers about various aspects of wellbeing dimensions put forward by the framework, such as access to amenities and services, relations of love and care, and levels of (dis)satisfaction with services and information. Also, it can be argued that paying attention to recognitional justice offers another pathway to capture people’s own perceptions of their material, social and personal wellbeing.

## Methods

In this article, we understand wellbeing as a people-centred process founded in a person’s socio-psychological perceptions and priorities. The qualitative research strategy deployed in this study therefore paid importance to people’s own perspectives and understandings, to grasping the meanings held by informants, and to the close involvement of the researcher with the informants (Bryman, 2012, p. 408).

Three groups of research participants were recruited for the study: migrant workers, volunteers involved in relief work during the first lockdown, and local self-government institution (panchayat) members. Primary data were collected through interviews. Ten migrant workers based in Kerala during the first lockdown, five volunteers from two voluntary initiatives, and three ward members and block panchayat members were interviewed (see Table 2). This article will refer to ward members and block members as panchayat members.

**Table 2:** Overview of research participants.

Research participant group	Number of participants	Age	Gender	Livelihood
Migrant workers	10	Between 20–40	Male (10)	Painter, saloon worker, road construction worker, bike mechanic, daily wage workers.
Local government members	3	Between 30–60	Female (1) Male (2)	Ex-members of local government.
Volunteers	5	Between 20–30	Female (2) Male (3)	Graduate and Post Graduate students, PhD Scholar, Development sector professionals

At the time of the interviews, the migrant workers were either in Kerala or had returned to their home state and were waiting to return to Kerala. Most of them had been in Kerala for a long time, that is, more than five years. The panchayat members held their positions during the first lockdown and not necessarily during the time of interviews as some of them had lost the elections conducted after the first lockdown. Interviews were taken online as the lead author could not travel to Kerala due to a new lockdown following the second wave of COVID-19. The duration of the interviews varied from one hour to two hours. According to feasibility and preference of the research participants, Zoom, WhatsApp or telephonic calls were used to conduct the interviews. Semi-structured interview schedules were facilitated by the use of open-ended questions. Different question schedules were used for each group of research participants. The access to migrant workers was obtained through ‘Lets Reach Out Kerala’ (LROK), a project under the National Health Mission, Ernakulam, Kerala, where the lead author was a Core Team Member. LROK provided psycho-social support to migrant workers, who were contacted by its volunteers over phone. The permission to access the contact numbers of migrant workers was granted by the Project Directors.

Convenience and snowball sampling were used to recruit participants. The call reports of LROK were studied and a shortlisting was done based on the issues that had been reported. Ten migrant workers from this shortlist were contacted and all of them agreed to participate. These call reports are used in the study to substantiate the data collected through the interviews. The volunteers were contacted based on their work with various initiatives. Contacts of the panchayat members were received via a gatekeeper.

Data collection took place in the summer of 2021, after ethics review approval was obtained. All participants provided informed consent either verbally or written. In this article, their names have been changed to protect their identity and integrity. As a Core Team Member of LROK, the lead author was involved with the three research participant groups and gained experience in working with the migrant workers. Through these she became aware of their social context and vulnerabilities, and she remained highly sensitive towards them throughout the research.

## Material, relational and subjective dimensions of wellbeing

The concerns of material, relational and subjective wellbeing are practical welfare and standard of living, social and human spheres, and people’s own perception of their material, social and human positions as well as their cultural values, ideologies and beliefs. Each of these concerns comprise a range of aspects. This article engages with some of the selected aspects from these dimensions: employment and livelihoods, access to services and amenities, relations of love and care, migrants’ cultural, political and social identities, and their perceptions of safety, trust and confidence. The article focuses on the subjective side of these dimensions—people’s own perceptions of their material, social and human wellbeing, as discussed in the following sections.

### Employment and livelihood: ‘I was sitting in my room with no work for around 40 days’

There is a high influx of inter-state migrant workers in Kerala, and they are employed in various sectors of the economy, including hotels and restaurants, construction, plumbing, painting, and various forms of self-employment (Sasidevan et al., 2021, p. 71). With all shops, businesses and construction projects shut during the COVID-19 lockdown, migrant workers were among the worst hit groups. With no jobs, they stayed indoors for a period of three months, which impacted them in many ways.

A major impact on migrant workers was the loss of livelihood and source of income (Jan Sahas, 2020, p. 6; Raju et al., 2021, p. 3). A considerable proportion of migrant workers’ incomes is normally sent to their families and, therefore, the loss of income meant not only a lack of money for their own expenses but also for their families back home, whose survival often partially or completely depended on these remittances. Those who had a safety net of savings resorted to that, while others resorted to borrowing money from employers, house owners or friends. In some instances, they even had to ask their families to send them money, thus resulting in reverse remittances. This caused additional stress for the families as this led to the depletion of whatever savings they might have had. Migrant workers also shared that their landlords or employers gave them money but not on credit or as a loan. Abdu said that his employer gave him an initial amount of Rs. 3000/- with a promise to give more money as needed in the future. While these gestures were deemed very helpful, they could only support the needs of stranded migrant workers themselves, nor their families back home. This remained a considerable cause of worry for many of them. These forms of support reveal a clear recognition of migrant workers’ material needs, even though this recognitional justice rarely included the needs of their kin back home.

It is worth noting that their jobs were never just a means to earn money. For many, having work meant being productive and engaging in activities they were already familiar with. We must also note the meaning that they attached to their work and how important it was to them. Most of them had been working all their lives and therefore staying indoors without work made little sense to them—they wanted to be productive again and as soon as possible. To evaluate the impact of this on their mental health requires a more detailed study but it is important to note that several migrant workers felt that being without employment caused them to worry (Sasidevan et al., 2021, p. 17, 27, 45, 56). Contrastingly, some migrant workers took this time as a vacation (The Week, 2020) they always wanted—a time to rest and a break from their daily routine of long working hours.

During the interviews with panchayat members, another impact concerned the available amenities at their places of stay. Mini and Yaser, both panchayat members, said that many migrant workers shared a single accommodation, often leading to crammed situations. But, due to the nature of their work, which involves different shifts, not all of them were generally present in the accommodation at any given time. Therefore, situations of pressure on amenities like water and electricity did not normally arise as a problem. But during lockdown, with all residents waiting indoors at the same time, pressure on such amenities became a problem, with house owners finding it difficult to address them. Also, space in these places of stay was deemed insufficient, posing a challenge to maintaining social distancing.

Lockdown thus impacted the main two reasons for migration to Kerala negatively—higher wages and availability of work. For many, however, losing jobs was not the only impact of the lockdown. Draining of their savings, falling into debts (Action Aid, 2020), stress at their places of stay, and feelings of unproductivity were other major impacts. Despite the uncertainty of the lockdown and the pain of being in a different state away from family, many migrant workers decided to stay back in Kerala because the state ensured more job security (Shibu, 2020; The Hindu, 2020). They were hopeful of resuming their work as soon as the lockdown was lifted, or at least of finding another job within Kerala. The migrant workers who stayed back said that they had started working again by the time of the interview, whereas a participant who had gone home said that he was currently unemployed and unable to return to Kerala. We now turn to how the lockdown impacted access to services and amenities.

### Access to services and amenities: ‘I received ration from the panchayat, but only once. It wasn’t sufficient to get through the lockdown’

Kerala has been known for its welfare schemes for migrant workers (Peter et al., 2020, p.1065). Migrant workers have access to the Public Distribution System (PDS) via ration shops, schools, Public Health Centres (PHC), and *anganwadis* (childcare centres). Along with these, lockdown saw tailor-made services emerge to cater to newly emerging needs during lockdown. The Community Kitchen was one of the main services set up at the time. Community Kitchens were kitchens put in place in each panchayat by *kudumbashree* members and the panchayats to prepare cooked meals for the socially and financially ‘downtrodden’, including migrant workers (Bechu, 2020). Many volunteers, mostly the youth, joined these kitchens providing various services. The migrant workers either collected packed meals from these kitchens or meals were delivered to their residences by volunteers, panchayat members or the police. A list with all such Community Kitchens was prepared and widely distributed by those involved in relief activities.

Even though the specific needs of migrant workers were recognised and included in such relief efforts, a major issue concerned the taste of the food. The meals were prepared in Kerala style with coconut oil, and they tasted very different from the food that migrant workers were used to, which was cooked with mustard oil, vegetable oil or sunflower oil. Normally, they would buy the raw ingredients and prepare their own food. There were instances when the migrant workers did not accept the packed meals delivered to them for this reason. A volunteer who had coordinated this food delivery with the panchayat member, and who was also a research participant, mentioned that such instances contributed to resentment from the wider public towards migrant workers. She added that the migrant worker had asked her ‘*at a difficult time like this, when I miss home so much, is it wrong to expect our food’*. Similar sentiments were shared by other migrant workers as well, saying that having their own cuisine, the closest thing to home, would be a relief, and that it was something they very much missed during the lockdowns. Kabir explained that he received the fat rice in his kit—‘*I don’t like it. I couldn’t eat it, but I tried to adjust’*.

During the lockdown, ration kits containing rice, oil, lentils, and sugar were distributed to the migrant workers. Rahim explained that he received vegetables, lentils, rice, and sugar from the panchayat. Interviews and call reports of LROK revealed that many migrant workers reported not having received these kits. Fasil described how he contacted the panchayat member who took his details and provided him with a few kilogrammes of rice. However, he received this only once and he did not get anything else like lentils or masalas with the rice. He was able to procure them from a nearby shop on credit as he had good relations with the shop owner. Ramzan stated that he had to make multiple calls to the local government bodies before he received food, and that too only once. He received wheat flour, lentils, onions, chillies, turmeric and sugar.

Another major service is the DISHA, a tele-helpline number for providing psycho-social support. This was another area in which the specific experiences of migrant workers were recognised and where concrete attempts were made to address them. The migrant workers were encouraged to call this helpline whenever they faced any difficulties. Since they spok different languages, this aspect was given attention to in terms of ensuring that the responders spoke multiple languages. They could call up and talk in the language they were comfortable with and not necessarily Malayalam, the official language of Kerala. The call reports of LROK revealed that many migrant workers were not aware of the helpline number, but were informed about it and encouraged by volunteers to call it in case of need. There were instances when the volunteers contacted the helpline on behalf of migrant workers and were assured help. The volunteers and migrant workers also contacted the District Labour Officers and Assistant Labour Officers for matters that needed more specific attention. The numbers of the labour offices were available online and could be accessed by the public.

We thus see that there were various provisions for amenities and services provided by the Kerala government, which reflected a considerable degree of attention to recognitional justice on its part. However, turning such recognition into appropriate action came with its own challenges as testified by the multiple issues of accessibility, in terms of knowledge of and access to help (as in the case of the helpline number and ration kits), and the suitability of provisions (as in the case of packed meals). It is also important to see how the migrant workers perceived the help handed out to them and why, as in the example shared by the volunteer, they declined some of the help offered.

### Relations of care and support: ‘I was able to buy things from a shop near me on credit as I know the shopkeeper’

The most important bond that migrant workers shared with each other was their sense of love and care for one another. From the interviews with volunteers and the call reports of LROK, it was clear that when a volunteer called to check on a migrant worker, they expressed concerns over their co-resident migrant workers too. Talking to one migrant worker was, therefore, like talking to the group staying with him. Sometimes they shared concerns for those they knew but who were staying in a different place or even a different district. Abhay, for example, was concerned about his friend who was living far from him and was suffering from throat pain and fever. He expressed this to the volunteer who contacted his friend and guided him in relation to the help available. Later on, Abhay was in touch with the volunteer for updates about his friend. A volunteer shared in the interview that they received many similar concerns about a fellow migrant worker or even a family member in a different state. The volunteer said that their initiative tried their best to address any concerns they received.

Another important relation was between the migrant workers and their employers. Unlike what happened in other states (Carswell et al., 2022), we learned from the interviews and call reports that many employers provided their employee migrant workers with accommodation, food and money. Many hotel owners let the migrant workers stay in the hotel premises itself and provided them with food. They were also able to resume their work in the hotels as soon as the lockdown restrictions were lifted. With accommodation and food taken care of, they were relieved from worrying about these and the lack of wages was, therefore, less of an immediate concern for them. Interestingly, the hotel employee migrant workers in the study did not know about the Community Kitchens. They mentioned that since they were receiving food from their employers, they did not have to rely on such government interventions. As one volunteer noted in one of his call reports*: “Once when I started talking about Community Kitchen, he said that they think they won’t be needed because they are free to ask anything at any time to their Ahmed Ikka”*. Ahmed, owner of Altaf Hotel in Aluva, provided food for 24 migrant workers including those who were not his employees. Wahim, a hotel owner in Kochi, gave Rs. 2000/- to his employees to buy rations and promised to give more money should the need arise.

Similarly, house owners also provided monetary and non-monetary help to their tenant migrant workers. On the government instruction not to collect rent from migrant tenants, many house owners did not ask for rent during the lockdown months. Some house owners provided their migrant tenants with vegetables and other rations. One of the call reports read: *“The house owner is providing them with food and is in constant contact with them”*. Another report stated: *“The house owner informed us that he has been giving them 3* *kgs of rice (for 8 ppl), dal and egg, but is not able to give out other supplies. He told me he is a senior citizen and helping as much as possible”*. It is important to note that not all employers and house owners were providing similar assistance. In some places, migrant workers feared eviction (Sasidevan et al., 2021, p. 55, 56), while in other instances, the employers and house owners ‘ghosted’ them, or disappeared, and thereafter avoided their workers’ attempts to get in contact with them (see also Carswell et al., 2022 for the neighbouring state of Tamil Nadu).

Another important relation that this study identified is between the migrant workers and local grocery store owners. These stores have customers from the local neighbourhoods where the owner tends to know his customers personally and sell goods on credit. This is based on the trust that payments will be made on a future date. Migrant workers who had been in Kerala for a longer time were also able to develop such relations, and they turned out to be very resourceful during the lockdown. Fasil explained in his interview that he had good relations with the grocer who sold things to him on credit, which was a great relief for him during that difficult period.

Through the lived experiences of migrant workers, we gained insight into the different relations of care and support that the migrant workers developed in Kerala. The relationships the migrant workers developed with their employers, house owners, other locals and each other meant that their needs were visible to and recognised by these different actors during the COVID-19 crisis, as became clear from the support offered to migrant populations during the lockdown. We now turn to the cultural, political and social identities aspect of wellbeing.

### Cultural, political, and social identities: ‘In reality, referring to us as guest workers hasn’t resulted in any difference’

While presenting the budget for the financial year 2018–2019 in February 2018, the then Finance Minister of Kerala Thomas Issac used the term ‘guest workers’ or ‘*atithi thozhilakilal*’ to refer to inter-state migrant workers (The New Indian Express, 2018). Though both government and ordinary citizens have been using the term to refer to migrant workers since then, the effects of this language shift continue to be highly debated among scholars. According to Kattakayam (2020) ‘guest’ is more welcoming than ‘migrant’ and in that way the ‘guest worker’s’ specific needs become recognised by the state and, hence, a responsibility of the state. However, Peter et al. (2020, p.1075) argue that this term furthers the process of ‘othering’ and presents them as less privileged than Keralites. We asked migrant workers how they felt about being called ‘guest workers’ and if the use of this term reflected any difference in terms of the treatment they received from Keralites. It is interesting to note that migrant workers interviewed in the study gave mixed responses to this. One of the ten migrant workers interviewed said that there were changes due to this usage and he felt that it was like accepting someone into a family. Another migrant worker opined that he saw no problem in being referred to as guest, but he did not see much difference in terms of how they were treated.

Kiran replied with a rhetorical question when he was asked, *“… bolne se kya hoga?”* (‘What will happen by just speaking?’). According to Rahim, “*Bolne se nahi hoga, thoda karne se hoga”* (‘Speaking will not do the work, actions will only work’). Another migrant worker opined that there should be corresponding actions from the government in terms of respectful salary and better working conditions. Interviews with volunteers and panchayat members gave a more positive picture. They felt that using ‘*atithi thozhilalikal*’ (‘guest workers’) was more welcoming and accommodating. They considered it a major shift from the use of ‘Bengali.’ Although this term referred to a person from the state of West Bengal, it was used as an umbrella term to refer to migrant workers irrespective of their home state and had a derogatory connotation. Thus, according to them, this shift was more dignified and was reflected in behavioural changes amongst the public, thus revealing a desire to disburse recognitional justice to what was clearly recognised as a vulnerable and often stigmatised group in the Kerala society.

Although there were only few material changes that followed the adoption of the ‘guest workers’ term, further attempts were made to shed more derogatory terms and attitudes towards migrant workers in the state. But, it is essential to note that not all migrant workers felt that way. Like Rahim, they wanted to see it materialised through tangible outputs. This is crucial from a policy planning perspective, as we will discuss below. While recognitional justice requires a shift at the level of recognition, it also needs to translate into meaningful actions to become truly transformational. We now move on to another aspect of wellbeing: migrant workers’ perceptions of safety.

### Perceptions of safety: ‘The lockdown went well. There was a feeling of security over Kerala’

For migrant workers, Kerala is a place of hope for better wages and improved standards of living. The by-product of this suddenly imposed lockdown was uncertainty of not knowing *when* they would be able to resume their work or *whether* they would be able to resume work at all. This section addresses migrants’ perceptions of safety with respect to income and job security. The effects of economic and non-economic losses of wellbeing are well-established, such as, for example, in the study of Bhola Slum dwellers who wished to return to their native land on the southern coast of Bangladesh after experiencing mental and physical ill health as a result of the move, environmental impacts and the living conditions in the slum (Ayeb-Karlsson, 2021, p. 352)

A major fear during the lockdown was the depletion of savings. Having no source of income, they had to meet their day-to-day expenses from the money they had saved. After keeping a minimal amount for their daily expenses, these migrant workers normally send most of their earnings back home, where it is used for meeting various expenses or saved. According to one of the volunteers, due to this, they did not have much money at their disposal. Many had to ask their family to send some money back to them. Some borrowed money from their friends or employers or house owners. This was one of the concerns they shared with the LROK volunteers. This uncertainty posed questions about their ability to realise their hopes and aspirations. They feared that not being able to work and earn money, having to live off savings or borrowed money, would result in their hard work of many years being reduced to nothing.

The feeling of safety or security that the migrant workers experienced can also be understood in relation to their (un)willingness to go back to their home states. Towards the end of the lockdown in May 2020, special *Shramik* (worker) trains were arranged to facilitate the travel of migrant workers back to their villages. Volunteers shared in their interviews that many migrant workers preferred to remain in Kerala rather than to go back to their home in the hope that they would be able to resume work once the lockdown was lifted. For them, Kerala was an assured source of employment, which their home villages were not. They feared that once they went home, they would be unable to return to Kerala or find work at home. They hoped, however, that if they remained in Kerala, they might be able to find work again. Rahul expressed that he had gone back to his village on a *Shramik* train. After the lockdown, he had been trying to return to Kerala for four months but he had been unable to do so. He feared that he would not be able to return at all and lose his job, which was key to his aspirations for a better life. We saw in the LROK call reports that the fear of draining savings, having no money with them and losing their jobs caused mental and emotional stress for the migrant workers. The interviewed migrant workers who stayed back in Kerala were able to resume their work or get another job soon after the lockdown. According to a study conducted by the Centre for Policy and Development Studies, only 4.5 lakh [450,000] migrant workers in Kerala returned to their native places while the rest of the migrant workers stayed back in Kerala (Centre for Policy and Development Studies, 2021 cited in Urban Update, 2021 & DNA, 2021).

Another reason why the migrant workers interviewed in this study felt safe in Kerala was related to the help they were receiving. According to one interviewee, he felt a sense of security in Kerala as he received help not just from the government but also from his employee and the locals. The migrant workers also spoke about the help that they received from the police, who did not just visit them in their residences to check up on them but they also delivered food kits. Thus, Kerala continued to be considered a place of earning and job security against the economic losses that COVID-19 and the lockdown brought. Along with this, the state was able to extend a perception of safety to its migrant worker population through the various psycho-social help and the support it extended. It is important to note that the panchayat members interviewed stated that their experiences with the flood responses had equipped them to respond better to the pressures of the pandemic and lockdown. They had a resident database of their panchayats from the flood response, which facilitated the need assessments that were carried out during the lockdown. They were unprepared during the first flood in 2018, but the disaster response to subsequent and smaller floods enabled them to gain experience in disaster preparedness and response. The volunteers also commented on the familiarity of the panchayat members with the migrant workers in their wards. This local knowledge about the presence and needs of migrant networks within panchayats formed the basis for the subsequent attempts at measuring out recognitional justice during the COVID-19 crisis. This brings us to the final aspect of wellbeing: trust and confidence.

### Trust and confidence: ‘Whenever I needed anything, I would call the volunteers and/or panchayat members’

In the Employment and Livelihood section, we mentioned Abdu, whose employer gave him Rs. 3000/- as an initial amount and agreed to help him more if needed. Another migrant worker shared that his employer gave him Rs. 2000/- for rations. The employees of Ahmed did not know about the Community Kitchen as their ‘Ahmed Ikka’ was taking care of their food requirements. In many instances, the employers provided food and/or accommodation to their employees. At uncertain times, such as during the pandemic, many migrant workers had their employer as their main safety net. Therefore, employers such as Ahmed were able to gain the trust or confidence of their workers, or they were able to act on such existing trust during this time of crisis.

At the same time, there were migrant workers with different experiences. In the initial days of the lockdown, Shivam’s contractor provided him with some rations. After a few weeks, the contractor said that he could no longer provide any more rations and a couple of days later, he was not even reachable on the phone. For Shivam, an assured source of help was suddenly gone. In instances like these, the trust that the employees had placed in their employers was broken.

Another important relation of trust and confidence was between migrant workers and volunteers. Through helping them, the volunteers gained the confidence of migrant workers. This was reflected in the interview with a participant who expressed how a *Didi* (elder sister in Hindi) used to help him out. She reached out to him initially as part of a voluntary initiative and was able to help him. He shared that when the need arose, he used to call the *Didi* rather than the panchayat member or his employer, as support from her was guaranteed.

Another relation of trust that this article identifies was between the migrant workers and the local shop owners. We saw in one of the earlier sections how Ramzan was able to procure rations from the local shop owner on credit when he did not receive them in his kits from the panchayat and did not have money to buy them himself. Selling on credit is a common feature of local shops, based on trust between the shop owner and the creditor. Migrant workers were also integrated into that system, and their needs were recognised by their local peers.

It is important to note that many migrant workers were confident about finding a job in Kerala after the lockdown. Adding to this, in many of the call reports, we noted how the migrant workers felt that they would be better off in Kerala during the lockdown than in their own home states. These are the main reasons for why they preferred to remain in Kerala. Through assured work, higher wages and various government schemes resulting in a better standard of living, Kerala had been able to build trust and confidence in migrant workers, and that they would be able to realise their aspirations of a better life. Reading this in tandem with perceptions of safety sheds light on both these subjective aspects of wellbeing.

So far, we have laid out six aspects of wellbeing across the three dimensions. We have seen the experiences of migrant workers during the first national lockdown and the various forms of help they received from actors such as the local government, house owners, employers, shopkeepers and voluntary initiatives. We also explored how migrant workers perceived their experiences at that time as well as the help they received or failed to receive. We will now discuss these findings and their relevance to policy making.

## Wellbeing and recognitional justice

The study revealed that migrants’ material wellbeing was severely affected during the pandemic and lockdowns. The loss of employment during the three lockdown months and beyond meant having little to no income. This posed a major crisis for the workers who depended on their daily wages to meet their expenses. Those who had savings resorted to it, but the length of the lockdown resulted in the rapid depletion of these savings. In some cases, this led to reverse remittance flows, which caused worry as it put strains on the families who depended on these remittances. Thus, all aspects of material wellbeing—income, employment, and levels of consumption—of the migrant workers as well as their families, were affected. Apart from this, the loss of employment also meant that they had to stay indoors and sit idle, which felt unusual. Being used to working daily, staying indoors with no work made little sense. The reduced material wellbeing caused fears, emotional and mental stress, thus affecting their subjective wellbeing. Regarding access to services and amenities, though efforts were made by the state and local governments to address these, this was not uniformly received by the migrant workers. While some were able to access the various provisions, others shared that they had not received anything due to a lack of information or that they were only one-time beneficiaries. For a state that has been commented on for its exceptional services to migrant workers, these experiences point to remaining issues to be addressed with respect to recognitional justice. Clearly, even much valued efforts can still result in feelings of being rendered invisible or neglected as migrant workers in the state.

The relational wellbeing of Kerala’s migrant workers during the lockdown was experienced through relations of love, care and support. The migrant workers did not just receive help from the government but also from other actors in Kerala. Our study identified these relations to be majorly with their employers or contractors, house owners, neighbours, local shop owners and volunteers. In many instances, employers and house owners provided free accommodation, food, and money. Shop owners sold them provisions on credit, while neighbours gave them food and other necessities, and pandemic response volunteers extended psycho-social support. Most times, the migrant workers even preferred these volunteer services to those of the panchayat members as they were available and effective. The study shows how these relations of love and care resulted in increased trust and confidence, as well as a sense of safety in Kerala during the lockdown. Thus, we see that key aspects of relational wellbeing resulted in an enhanced subjective sense of wellbeing.

The advantage of WeD is that it enables a subjective understanding of wellbeing. Its people-centred approach allows us, in this context, to examine how various government support measures aimed at migrant workers were perceived and experienced by them. Levels of relative (dis)satisfaction with information and services, which is an aspect of subjective wellbeing, must be understood as evaluation measures of success in the policies and support measures. Understanding these will help inform and improve future policies and measures for disaster preparedness and response. For instance, the way that migrant workers perceived being called *‘atithi thozhilalikal’* (‘guest workers’) was ambivalent. While the panchayat members and volunteers felt it was a step towards inclusion and positive change, many migrant workers felt that these changes in the language used to describe them were not necessarily translated into actions. Some reported preferring more effective action rather than a vocabulary change. Migrant workers had similar feelings related to the packed meals from the Community Kitchens. Though the aim was to address food shortages, the differences in cuisine led to its purpose largely being defeated. As a result, some migrant workers rejected the food packets altogether. This rejection was met with a public resentment towards them. Migrant workers described preferring voluntary services and initiatives over those of the government as they were more responsive and effective. For instance, a migrant worker explained calling a volunteer connection rather than a panchayat member in times of need as he knew what help to expect from each of them. This is very important from a policy practice perspective as it can help improve implementation on the ground.

From a recognitional justice standpoint, we evaluated various interventions and relief efforts by different actors, as well as their recognition and understanding of the specific needs of migrant workers in the state. While we found evidence of sensitivity towards migrant workers, we also identified challenges of turning such sensitivity into transformative policy practice and implementation. The use of the term ‘guest workers,’ for example, can be seen as a step towards enhancing visibility and social esteem, recognising sociocultural identities, and addressing institutional inequities. However, the subjective dimension of our wellbeing analysis revealed limitations in how the term was received. Another example relates to the distribution of cooked meals. While the distribution of such meals reflected attempts to include migrant workers in relief work, some of the effort was undone by a perceived lack of sensitivity towards migrant workers’ specific food habits. The provided food failed to recognise and integrate sociocultural identities and values into policy making, which are basic to recognitional justice.

While the material dimensions of disaster relief are common in development planning, we note, as White (2010) suggests, that the three dimensions of wellbeing are inter-related and co-constitutive. The subjective wellbeing of the migrant workers in our study was shaped by their experiences of material and relational wellbeing dimensions. Relations of love, care and support contributed to feelings of trust and confidence, while (in)access to services and amenities resulted in (dis)satisfaction with certain provisions. In some instances, the dissatisfaction with services even led to questioning their new Malayali migrant identity as guest workers. These research findings around both subjective wellbeing and recognitional justice can inform disaster preparedness, response and policy at planning and implementation levels. They can remind policy makers of the unique need to ensure integration of material, relational and subjective dimensions of wellbeing in disaster preparedness policies. Giving attention to the lived experiences and perceptions of beneficiaries facilitates ownership over local policies. It extends a feeling of being heard and included in the policies intended for them, which is vital for more successful and effective policy design as well as for the attainment of more meaningful recognitional justice for all—especially at times of crisis (Chu and Michael, 2019).

## Conclusion

This article investigated the wellbeing of migrant workers stranded in Kerala, India, during the first national COVID-19 lockdown of 2020. We drew on the three dimensions of wellbeing (material, relational and subjective) in the WeD approach. Interviews with migrant workers in Kerala during the lockdown as well as with panchayat members and volunteers involved in relief activities served to be incredibly valuable. The Kerala government was able to deliver its programmes for the migrant workers as indicated by various scholars (Peter et al., 2020, p. 1065; Chathukulam and Tharamangalam, 2020; Rao et al., 2020, p. 1658; Dutta and Fischer, 2020, p. 8). However, our analysis also points out that while the government interventions focused on material wellbeing dimensions, it is essential to integrate these with the relational and subjective dimensions of wellbeing. This is particularly the case during a time of crises, when we must understand the meanings that migrant workers attached to their work, the nature of their specific needs, and their perceptions of support and relief work. A crisis as extensive as the pandemic severely impacted the hopes, fears, and aspirations of migrant workers across India. The research findings coming out of this study will support the Indian and other governments to improve disaster policy planning and practice. A more wholesome wellbeing analysis, inclusive of perceptions of material, social and human positions, along with a more holistic and inclusive policy approach to wellbeing and recognitional justice will help ensure that Kerala’s new ‘guest workers’ do not remain mere words but translate into meaningful reforms.

## Data Availability

The datasets generated during and/or analysed during the current study are not publicly available due to data protection regulations but may be available from the corresponding author on reasonable request.

## Footnotes

1 We acknowledge the problematic post-colonial framing around the term ‘developing countries’ (see, for example, Ayeb-Karlsson 2020) but as this is the language used in the WeD framework it will appear in this article.
